# Kidney replacement therapy and mortality in metformin-associated lactic acidosis with acute kidney injury (2015–2021): a multi-year cohort study

**DOI:** 10.1080/0886022X.2025.2586333

**Published:** 2025-11-24

**Authors:** Sarassawan Kananuruks, Ekthanat Akkarakanoksin, Thanachai Panaput, Surasak Faisatjatham, Suphanan Phisalwut, Arkom Nongnuch

**Affiliations:** ^a^ Division of Nephrology, Department of Medicine, Khon Kaen Hospital, Khon Kaen, Thailand; ^b^ Department of Medicine, Khon Kaen Hospital, Khon Kaen, Thailand; ^c^ Division of Nephrology, Department of Medicine, Faculty of Medicine, Ramathibodi Hospital, Mahidol University, Bangkok, Thailand

**Keywords:** Metformin-Associated Lactic Acidosis, MALA, acute kidney injury, kidney replacement therapy (KRT), dialysis, mortality

## Abstract

Metformin-associated lactic acidosis (MALA) is a life-threatening complication. Treatment of MALA includes supportive care and dialysis. However, the role of kidney replacement therapy (KRT) in MALA is controversial, this study aimed to compare patient and kidney outcomes between MALA–acute kidney injury (AKI) patients receiving KRT (KRT group) vs. supportive care (supportive care group). A cohort study was conducted on 143 adult patients with MALA–AKI from 1 January 2015 to 31 December 2021. The primary outcome was the patient mortality rate and predictive factors of mortality, while the secondary outcomes were kidney survival. The overall mortality rate in our MALA–AKI cohort was 20.3% (29 of 143 patients). The KRT group had a lower mortality rate compared with the supportive care group (16% vs. 28.6%, *P* = 0.006). Among patients who received dialysis, hemodialysis (HD) was associated with a lower mortality rate compared with acute peritoneal dialysis (APD) (8.9% vs. 30.8%, *P* = 0.044). Patients who received late KRT (≥6 h) had a trend toward higher mortality compared with those who received early KRT (<6 h). Independent predictors of mortality were oliguria (HR 8.59, *P* = 0.002), prolonged prothrombin time (HR 1.20, *P* = 0.001) and higher Sequential Organ Failure Assessment (SOFA) scores (HR 1.26, *P* = 0.006). MALA–AKI was associated with high mortality, and KRT reduced mortality rate when compared with supportive care. HD was the most effective treatment in MALA–AKI, early KRT initiation (<6 h) showed a trend toward lower mortality and oliguria, prolonged prothrombin time, and higher SOFA scores were identified as prognostic markers.

## Graphical Abstract

**Figure:**
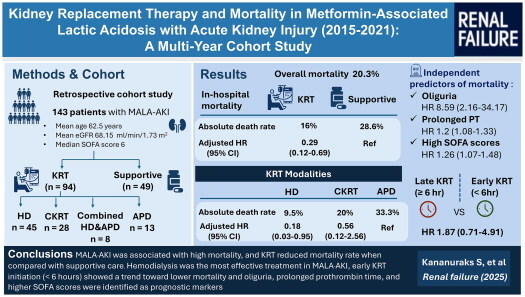

## Background

Metformin, a widely utilized and first line of oral antidiabetic medication, belongs to the biguanide class. The primary mechanism to lower glucose levels involves reducing glucose production in the liver and enhancing glucose usage in peripheral tissues [1,2]. Metformin is associated with a decrease in cardiovascular disease and mortality rates. This medication demonstrates efficacy and has economic value [3–5]. Nevertheless, the serious condition known as metformin-associated lactic acidosis (MALA) may arise in individuals with predisposing conditions like renal insufficiency, liver disorders, congestive heart failure, or hypotension [6–9]. Despite its relatively low incidence rate, ranging from 1 to 80 cases per 100,000 patients/year [6,7,10], the mortality associated with MALA is significantly high, with reported rates of 30-50% [7,9,11–13].

The cornerstone of initial management for MALA consists of resuscitation and providing supportive care. Kidney replacement therapy (KRT) may be considered in the treatment of MALA to eliminate metformin, and correction of acidemia and electrolyte abnormalities. However, limited studies are available on the benefits of KRT in MALA patients with acute kidney injury (MALA–AKI) [14]. Recent studies have shown that KRT improves survival rate [13,15–17], although some studies indicate that improvements may not be observed [11]. Studies have shown that high serum lactate levels, low pH, reduced prothrombin activity, and high Acute Physiology and Chronic Health Evaluation II scores are associated with an increased mortality rate [11,13,18–20].

This study aimed to compare patient and kidney outcomes between MALA–AKI patients receiving KRT (KRT group) versus those receiving supportive care (supportive care group), and to evaluate prognostic factors for mortality in these patients.

## Materials and methods

### Study population

The study was conducted at the inpatient Department of Medicine, Khon Kaen Hospital, Khon Kaen, Thailand, between 1 January 2015 and 31 December 2021. The inclusion criteria for our study were 18 years of age or older, having type 2 diabetes, receiving metformin treatment, and having MALA–AKI. Lactic acidosis is defined by lactate concentration higher than 5 mmol/L, arterial pH less than 7.35, and bicarbonate level less than 22 mmol/L. According to Kidney Disease: Improving Global Outcome (KDIGO) 2012 guidelines, AKI was defined by an increase in serum creatinine ≥0.3 within 48 h or an increase in serum creatinine ≥1.5 times baseline value within the prior 7 days or urine volume <0.5 mL/kg/h for 6 h [21]. Exclusion criteria included metformin overdose in the context of a suicide attempt, the presence of ketoacidosis, or the use of other drugs known to cause lactic acidosis. The study was approved by the Institutional Review Board of Khon Kaen Hospital (KEXP65010). All patient data were fully anonymized, and no identifying details of individual participants are included in this manuscript.

### Statistical analysis

The characteristics of the patients, including demographic data, dialysis information, and laboratory test findings, were reported. For continuous variables, the mean and standard deviation (SD) or the median and interquartile ranges were presented according to data as normal distribution or non-normal distribution respectively. Continuous variables were compared with the Student’s *t*-test (for normal distribution) or the Mann–Whitney *U* test (for non-normal distribution). Absolute values and percentages were used to report categorical data, which were compared via Fisher’s exact test. Analysis of the mortality rate in hospital among patients with MALA–AKI, in the KRT group compared to the supportive care group, using the log-rank test and the findings were illustrated through the Kaplan–Meier survival curve. Analysis of factors affecting mortality in hospitalized patients with MALA–AKI using multivariate Cox regression analysis, presented by hazard ratio (HR) and 95% confidence interval (CI). Analyses were done with Stata version 14.0. Statistical significance was established at *P* < 0.05, with all probability tests reported being two-sided.

## Results

**Figure 1.**
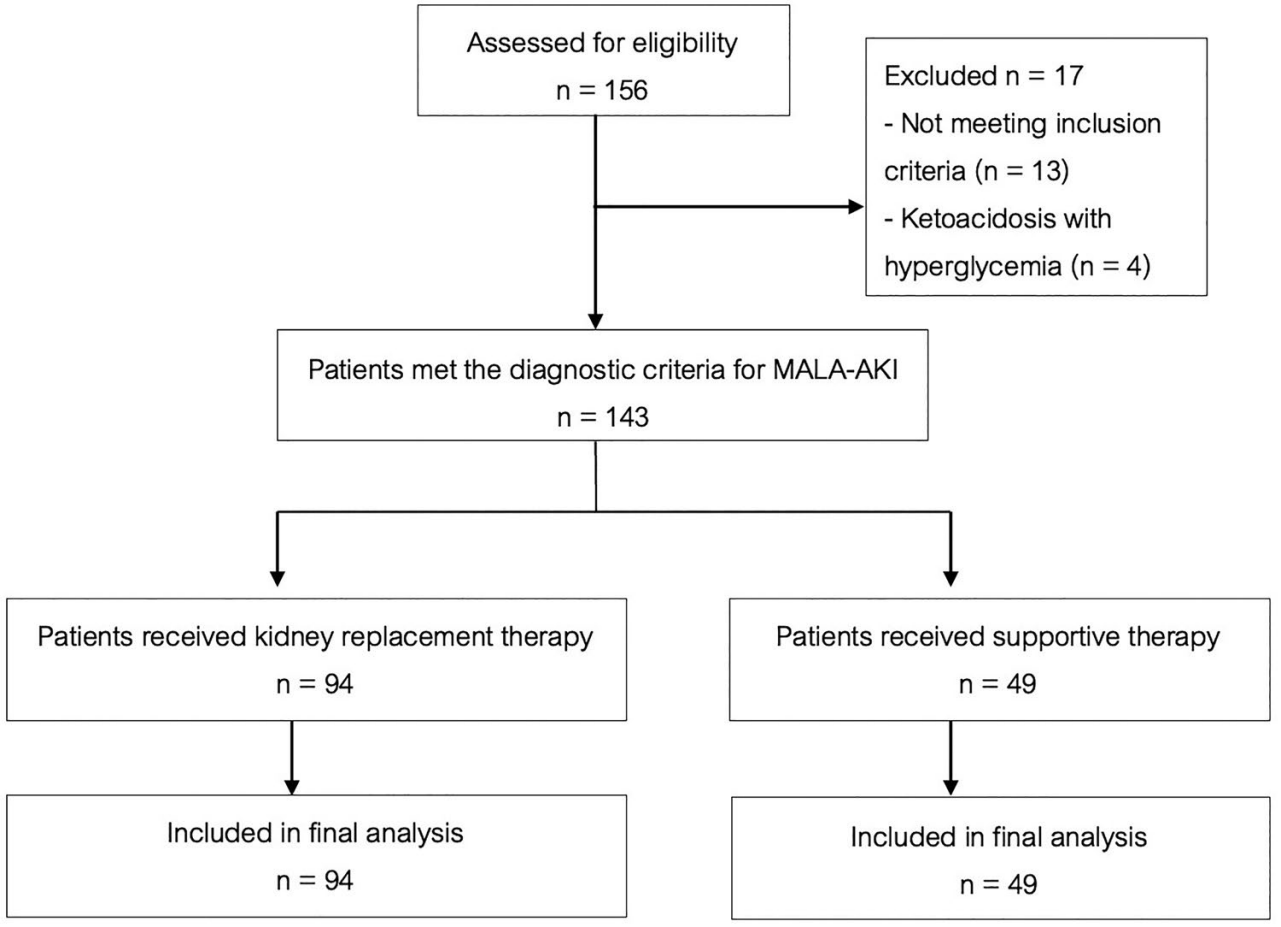
A flowchart of patients with MALA–AKI is included in this study.

A total of 143 patients were included in this study (Figure 1). 94 (65.73%) patients received KRT and 49 (34.27%) received supportive care. The mean age was 62.5 years, with females constituting 57.3% and 31.5% having chronic kidney disease. The mean estimated glomerular filtration rate (eGFR) before the onset of MALA–AKI was 68.15 mL/min/1.73 m^2^. Most of patients had AKI stage 3 (88.1%). The median dose of metformin received by patients was 2 g/day, and 18.2% of patients received an inappropriate dosage of metformin. The median Sequential Organ Failure Assessment (SOFA) score was 6, and 59.4% of patients received vasoactive support. There was no significant statistical difference in baseline characteristics between the two groups, except that patients who received KRT had more respiratory failure (*P* = 0.026) and more vasoactive therapy (*P* = 0.001). Moreover, patients in the KRT group were significantly more likely to receive treatment in the intensive care unit than those in the supportive care group (*P* < 0.001). The laboratory results of both groups did not differ significantly, except for the bicarbonate levels <5 mmol/L, which were significantly higher in the KRT group (*P* = 0.024) (Table 1). The median time to initiation of dialysis in the KRT group was 8 h.

**Table 1.** Baseline characteristics of patients with MALA–AKI.

Characteristics	All patients (*n* = 143)	KRT (*n* = 94)	Supportive treatment (*n* = 49)	*P* value
Age (years)	62.5 ± 9.8	62.0 ± 9.0	63.3 ± 11.1	0.471
Male sex, *n* (%)	61 (42.7)	44 (46.8)	17 (34.7)	0.164
Comorbidities, *n* (%)				
Chronic kidney disease	45 (31.5)	27 (28.7)	18 (36.7)	0.328
Chronic respiratory disease	3 (2.1)	2 (2.1)	1 (2.0)	0.973
Cardiovascular disease	18 (12.6)	11 (11.7)	7 (14.3)	0.668
Cancer	4 (2.8)	3 (3.2)	1 (2.0)	0.692
Baseline Cr (mg/dL)	1.04 ± 0.35	1.01 ± 0.29	1.11 ± 0.43	0.126
Baseline eGFR (mL/min/1.73 m^2^)	68.15 ± 22.68	70.40 ± 22.54	63.84 ± 22.56	0.101
AKI stage, *n* (%)				0.459
1	5 (3.5)	3 (3.2)	2 (4.1)	
2	12 (8.4)	6 (6.4)	6 (12.2)	
3	126 (88.1)	85 (90.4)	41 (83.7)	
Metformin dosage (g/day), *n* (%)				0.082
>2	25 (17.5)	20 (21.3)	5 (10.2)	
1–2	82 (57.3)	55 (58.5)	27 (55.1)	
<1	36 (25.2)	19 (20.2)	17 (34.7)	
Appropriateness of metformin use, *n* (%)	117 (81.8)	80 (85.1)	37 (75.5)	0.158
Other drugs, *n* (%)				
ACEI/ARBs	79 (55.2)	55 (58.5)	24 (49.0)	0.277
NSAIDs	3 (2.1)	1 (1.1)	2 (4.1)	0.232
Diuretics	11 (7.7)	5 (5.3)	6 (12.2)	0.140
SOFA score	6 (5,8)	6 (5,8)	5 (4,7)	0.166
Vasoactive therapy, *n* (%)	85 (59.4)	65 (69.2)	20 (40.8)	0.001
Symptoms, *n* (%)				
Alteration of consciousness	22 (15.4)	14 (14.9)	8 (16.3)	0.822
GI symptoms	118 (82.5)	79 (84.0)	39 (79.6)	0.506
Oliguria/anuria	37 (25.9)	20 (21.3)	17 (34.7)	0.082
Respiratory failure	88 (61.5)	64 (68.1)	24 (49.0)	0.026
Ward, *n* (%)				<0.001
MICU	47 (32.9)	44 (46.8)	3 (6.1)	
Medicine	96 (67.1)	50 (53.2)	46 (93.9)	
*Initial laboratory investigations*				
BUN (mg/dL)	68.7 ± 23.6	68.1 ± 21.4	69.7 ± 27.4	0.703
Creatinine (mg/dL)	8.61 ± 3.73	8.71 ± 3.55	8.41 ± 4.08	0.656
eGFR (mL/min/1.73 m^2^)	8.19 ± 8.94	7.75 ± 7.62	9.01 ± 11.09	0.425
pH	7.04 ± 0.19	7.03 ± 0.19	7.07 ± 0.19	0.173
pH, *n* (%)				0.102
<7	54 (37.8)	40 (42.6)	14 (28.6)	
≥7	89 (62.2)	54 (57.5)	35 (71.4)	
Bicarbonate (mmol/L)	5.57 ± 3.34	5.26 ± 3.34	6.17 ± 3.29	0.125
Bicarbonate (mmol/L), *n* (%)				
<5	77 (53.9)	57 (60.6)	20 (40.8)	0.024
5–15	66 (46.2)	37 (39.4)	29 (59.2)	
Lactate (mmol/L)	18.14 ± 7.29	18.87 ± 7.29	16.73 ± 8.63	0.119
Lactate (mmol/L), *n* (%)				0.138
<10 mmol/L	27 (18.9)	14 (14.9)	13 (26.5)	
10–20 mmol/L	59 (41.3)	38 (40.4)	21 (42.9)	
>20 mmol/L	57 (39.9)	42 (44.7)	15 (30.6)	
PT (s)	17.69 ± 5.09	17.60 ± 4.51	17.85 ± 6.07	0.796
aPTT (s)	43.34 ± 20.58	41.97 ± 14.14	45.87 ± 28.97	0.307
White blood cell count (/μL)	17,006.5 ± 6780.82	17,487.55 ± 7398.19	16,083.67 ± 5353.01	0.241
Hemoglobin (g/dL)	9.76 ± 1.90	9.81 ± 1.98	9.67 ± 1.74	0.692
Platelet count (10^3^/μL)	275 ± 92	268 ± 87	288 ± 101	0.337

eGFR = estimated glomerular filtration rate; AKI = acute kidney injury; ACEIs = angiotensin-converting enzyme inhibitors, ARBs = angiotensin II receptor antagonists, NSAIDs = non-steroidal anti-inflammatory drugs; SOFA score = Sequential Organ Failure Assessment Score; MICU = medical intensive care unit; BUN = blood urea nitrogen; PT = prothrombin time; aPTT = activated partial thromboplastin time.

**Figure 2.**
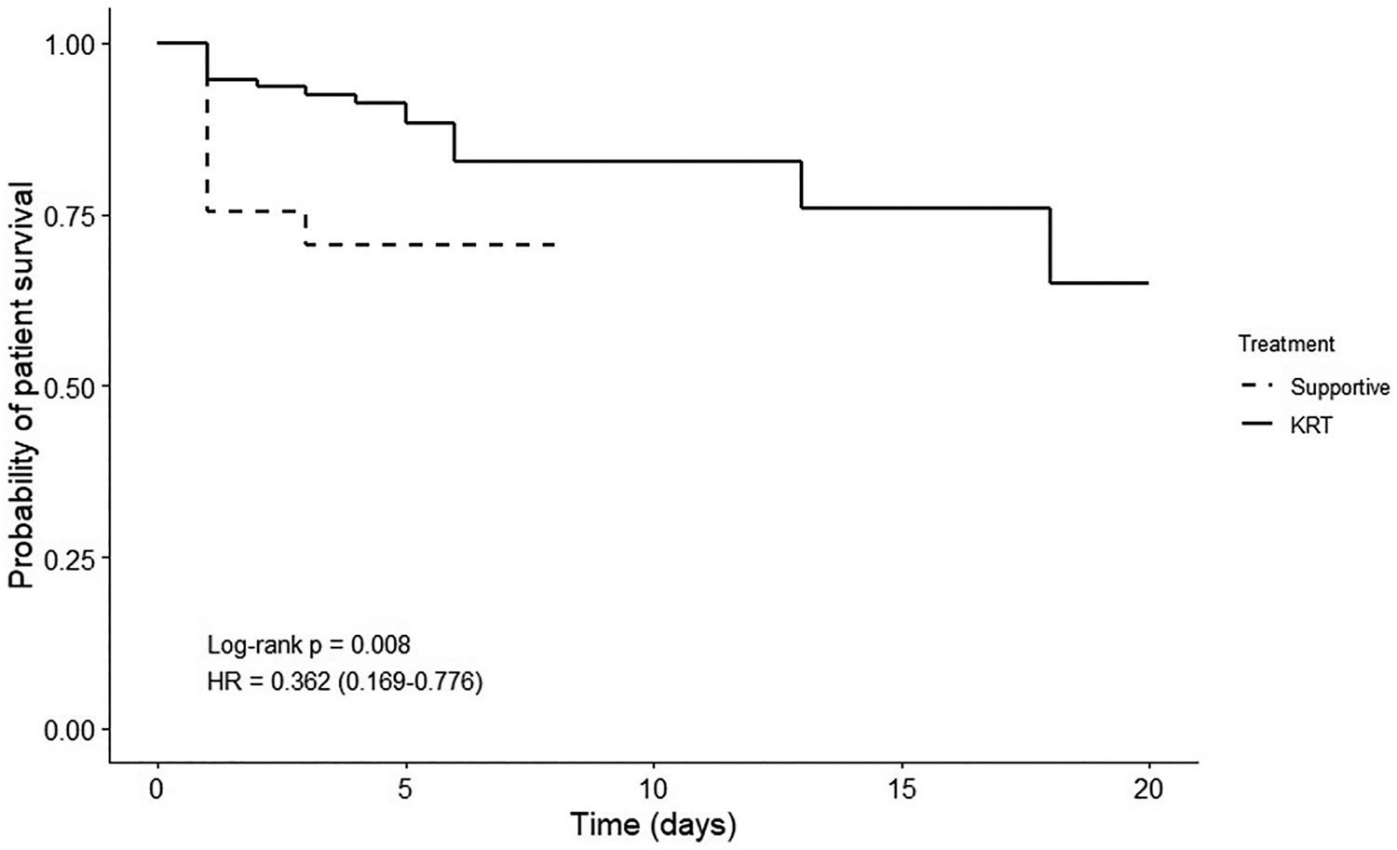
The Kaplan–Meier survival function for patients with MALA–AKI compared in the KRT group vs in the supportive care group.

The primary results of the study found that patients with MALA–AKI had an overall mortality rate of 20.3%. Moreover significantly lower mortality was observed in the KRT group compared to the supportive care group (16.0% vs 28.6%, *P* = 0.016). The KRT group was significantly associated with lower mortality than the supportive care group, with an adjusted HR of 0.29 (95% CI 0.12–0.69; *P* = 0.005) (Table 2 and Figure 2). Approximately 82.8% of patients with MALA–AKI died within the first 5 days of hospitalization, and 93.1% died within the first seven days. However, the recovery rate of kidney function did not differ significantly in both groups (*P* = 0.874) (Table 2).

**Table 2.** Mortality and kidney recovery in patients with MALA–AKI treated with KRT vs. supportive care.

Treatment outcomes	All patients (*n* = 143)	KRT (*n* = 94)	Supportive treatment (*n* = 49)	Univariate analysis		Multivariate analysis		
				Crude HR (95% CI)	*P* value	Adjusted HR (95% CI)^a^		*P* Value
Primary outcome								
In-hospital mortality, *n* (%)	29 (20.3)	15 (16.0)	14 (28.6)	0.39 (0.18–0.83)	0.016	0.29 (0.12–0.69)		0.005
Secondary outcome								
Renal recovery, *n* (%)^b^	114 (100)	79 (100)	35 (100)	0.92 (0.81–1.14)	0.874	–	–	–

^a^ The factors adjusted in the multivariate analysis were SOFA score and respiratory failure.

^b^All survivors recovered kidney function by discharge.

The factors associated with higher hospital mortality rates from the univariate analysis include altered level of consciousness, oliguria/anuria, respiratory failure, lower pH level, lower bicarbonate level, higher lactate level, longer prothrombin time, lower hemoglobin level, higher SOFA score, and vasoactive therapy (Table 3).

**Table 3.** The factors associated with the hospital mortality rate in patients with MALA–AKI.

Characteristics	All patients (*n* = 143)	Survivors (*n* = 114)	Non-survivors (*n* = 29)	Univariate analysis		Multivariate analysis	
				Crude HR (95% CI)	*P* value	Adjusted HR (95% CI)	*P* value
AKI stage, *n* (%)				0.81 (0.40–1.66)	0.57	–	–
1	5 (3.5)	4 (3.5)	1 (3.4)				
2	12 (8.4)	8 (7.5)	4 (13.8)				
3	126 (88.1)	102 (89.5)	24 (82.8)				
Symptoms, *n* (%)							
Alteration of consciousness	22 (15.4)	9 (7.9)	13 (44.8)	5.00 (2.40–10.43)	<0.001	–	–
GI symptoms	118 (82.5)	95 (83.3)	23 (79.3)	0.79 (0.32–1.94)	0.609	–	–
Oliguria/anuria	37 (25.9)	18 (15.8)	19 (65.5)	5.95 (2.75–12.88)	<0.001	8.59 (2.16–34.17)	0.002
Respiratory failure	88 (61.5)	61 (53.5)	27 (93.1)	8.16 (1.94–34.35)	0.004	–	–
Appropriateness of metformin use, *n* (%)	117 (81.8)	93 (81.6)	24 (82.8)	1.00 (0.38–2.64)	0.99	–	–
BUN (mg/dL)	68.65 ± 23.55	69.82 ± 23.50	64.03 ± 23.57	0.99 (0.98–1.01)	0.367	–	–
Creatinine (mg/dL)	8.61 ± 3.73	8.93 ± 3.67	7.35 ± 3.78	0.92 (0.83–1.02)	0.10	–	–
eGFR (mL/min/1.73 m^2^)	8.19 ± 8.94	7.57 ± 8.49	10.59 ± 10.36	1.02 (0.99–1.06)	0.138	–	–
pH	7.04 ± 0.19	7.06 ± 0.18	6.97 ± 0.19	0.12 (0.02–0.74)	0.022	–	–
pH, *n* (%)							
pH < 7	54 (37.8)	38 (33.3)	16 (55.2)	2.10 (1.01–4.37)	0.047	–	–
pH ≥ 7	89 (62.2)	76 (66.7)	13 (44.8)	1			
Bicarbonate (mmol/L), *n* (%)	5.57 ± 3.34	5.88 ± 3.37	4.37 ± 2.97	0.86 (0.74–0.98)	0.032	0.85 (0.70–1.04)	0.113
Bicarbonate, *n* (%)							
<5 (mmol/L)	77 (53.8)	56 (49.1)	21 (72.4)	2.46 (1.09–5.56)	0.031	–	–
≥5 (mmol/L)	66 (46.2)	58 (50.9)	8 (27.6)	1			
Lactate (mmol/L)	18.14 ± 7.81	17.08 ± 7.39	22.30 ± 8.17	1.07 (1.02–1.11)	0.003	–	–
Lactate (mmol/L), *n* (%)							
<10 mmol/L	27 (18.8)	24 (21.1)	3 (10.3)	1	–		
10–20 mmol/L	59 (41.3)	51 (44.7)	8 (27.6)	1.24 (0.33–4.68)	0.75	–	–
>20 mmol/L	57 (39.9)	39 (34.2)	18 (62.1)	3.09 (0.91–10.52)	0.07	–	–
PT (s)	17.69 ± 5.09	16.56 ± 3.63	21.57 ± 7.17	1.09 (1.04–1.13)	<0.001	1.20 (1.08–1.33)	0.001
Hemoglobin (g/dL)	9.77 ± 1.90	9.98 ± 1.83	8.91 ± 1.95	0.80 (0.67–0.96)	0.015	0.75 (0.52–1.07)	0.116
SOFA	6 (5,8)	6 (4.25,7)	9 (7,11)	1.59 (1.38–1.83)	<0.001	1.26 (1.07–1.48)	0.006
Vasoactive therapy, *n* (%)	85 (59.4)	58 (50.9)	27 (93.1)	8.81 (2.09–37.15)	0.003	–	–
Ward, *n* (%)				1.26 (0.57–2.78)	0.566	–	–
Medicine	47 (32.9)	38 (33.3)	76 (66.7)				
MICU	96 (67.1)	9 (31.0)	20 (69.0)				
Time interval before KRT (h)	8 (4,14)	7 (3.5,15)	11 (4,14)	1.00 (0.96–1.05)	0.84	–	–
Time interval before KRT, *n* (%)							
<6 h	38(26.57)	33(28.95)	5(17.24)	1			
≥6 h	105(73.43)	81(71.05)	24(82.76)	1.87 (0.71–4.91)	0.203	–	–
Mode, *n* (%)^a^							
HD	45 (31.5)	41 (36.0)	4 (13.8)	0.21 (0.05–0.86)	0.030	0.18 (0.03–0.95)	0.044
CKRT	28 (19.6)	22 (19.3)	6 (20.7)	0.53 (0.15–1.89)	0.327	0.56 (0.12–2.56)	0.457
Combined HD and APD	8 (5.6)	7 (6.1)	1 (3.4)	0.18 (0.02–1.75)	0.141	0.36 (0.03–3.71)	0.392
APD	13 (9.1)	9 (7.9)	4 (13.8)	1		1	

AKI = acute kidney injury; BUN = blood urea nitrogen; eGFR = estimated glomerular filtration rate; PT = prothrombin time; SOFA score = Sequential Organ Failure Assessment Score; MICU = medical intensive care unit; KRT = kidney replacement therapy; HD = hemodialysis; CKRT = continuous kidney replacement therapy; APD = acute peritoneal dialysis.

^a^ HD is defined as patients who received only hemodialysis throughout their treatment. CKRT is defined as patients who received only continuous KRT throughout their treatment. Combined HD and APD is defined as patients who initially received temporary acute peritoneal dialysis but were transitioned to HD due to APD failure. APD is defined as patients who received only acute peritoneal dialysis throughout their treatment.

**Figure 3.**
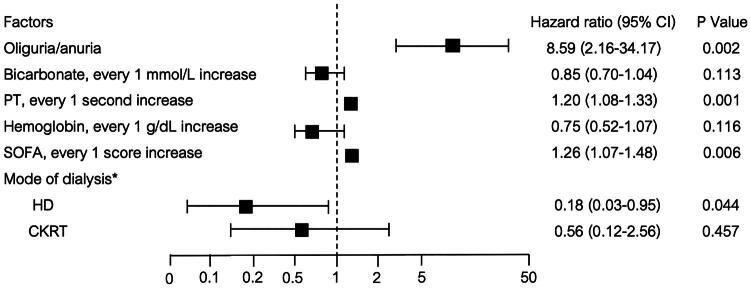
HR for hospital mortality in patients with MALA–AKI which were adjusted for oliguria/anuria, bicarbonate, PT, hemoglobin, SOFA score and mode of dialysis. *Mode of dialysis had APD as reference. PT = prothrombin time; SOFA score = Sequential Organ Failure Assessment Score; HD = hemodialysis; CKRT = continuous kidney replacement therapy.

**Figure 4.**
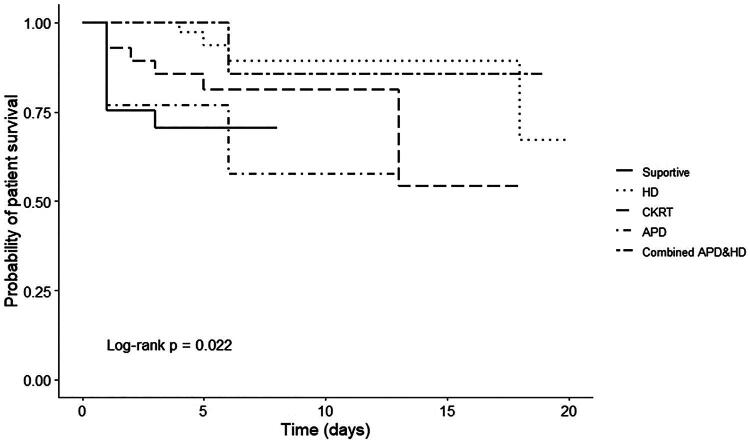
The Kaplan–Meier survival function for patients with MALA–AKI compared by treatment. HD is defined as patients who received only hemodialysis throughout their treatment. CKRT is defined as patients who received only continuous kidney replacement therapy throughout their treatment. Combined HD and APD is defined as patients who initially received temporary APD but were transitioned to HD due to APD failure. APD is defined as patients who received only acute peritoneal dialysis throughout their treatment.

From the multivariate Cox regression analysis, oliguria/anuria (HR = 8.59; 95% CI 2.16–34.17; *P* = 0.002), longer prothrombin time (HR = 1.20; 95% CI 1.08–1.33; *P* = 0.001) and higher SOFA score (HR = 1.26; 95% CI 1.07–1.48; *P* = 0.006) were significantly associated with an increase in-hospital mortality. (Table 3 and Figure 3). KRT via hemodialysis (HD) had the lowest mortality rate at 8.9% (HR = 0.18; 95% CI 0.03–0.95; *P* = 0.044), followed by continuous kidney replacement therapy (CKRT) at 21.4% (HR = 0.56; 95% CI 0.12–2.56; *P* = 0.457) compared with acute peritoneal dialysis (APD), which had a mortality rate of 30.8% (Table 3 and Figure 3). The proportion of survivors until hospital discharge stratified by dialysis modality among patients with MALA–AKI was illustrated in Figure 4.

## Discussion

In this study, patients with MALA–AKI had an overall mortality rate of 20.3%, which is comparable to previous studies, ranging from 21% to 33% [9,13,15,20,22]. Most patients (88.1%) presented with AKI stage 3. Additionally, 18.2% of patients were found to have received an inappropriate dosage of metformin based on the eGFR-dosing algorithm, which is lower than the percentage reported in the study by Yeh et al. where 42.5% of patients received an inappropriate dosage [19]. Although receiving an inappropriate dosage of metformin in both studies did not significantly impact mortality rates, this may imply that MALA occurred despite prescribing the appropriate dosage. Our study showed that KRT reduced the mortality rate in patients with MALA–AKI compared to supportive care by 71%. Most patients died from complications of MALA within the first 5 days, consistent with findings from previous studies [13]. According to the survival analysis, patients who received KRT had a lower in-hospital mortality rate over time. There are two patients supposed to be non-KRT related deaths on days 13 and 18, respectively. The first patient received KRT until creatinine and lactate levels normalized, but later developed colonic obstruction from recurrent cervical cancer and subsequently died in the hospital. The second patient, after treatment for MALA, developed a complication of E. coli MDR urinary tract infection that led to death. Both patients died from other complications not directly related to MALA. Despite that, the KRT group had more patients with acidemia, respiratory failure, received more vasoactive support, and had a higher rate of ICU admissions; these benefits highlight the important role of KRT in patients with MALA–AKI. However, the recovery rate of kidney function did not differ significantly between the two groups, in which all patients who survived subsequently recovered kidney function.

The optimal timing for initiating KRT in MALA–AKI remains a subject of investigation. Prior research has suggested that delaying KRT beyond 6 h is associated with increased mortality [13]. In our cohort, while we observed a trend toward lower mortality in patients receiving ‘early’ KRT (within 6 h of admission), this difference did not reach statistical significance. This result may be explained by the fact that most of our patients (70%) received KRT relatively promptly, within 12 h of admission, potentially blunting the statistical power to detect a difference between the groups. Nevertheless, the pathophysiological rationale for prompt intervention is strong. Delaying dialysis prolongs exposure to toxic levels of metformin and lactate. Since KRT effectively removes both substances and corrects various physiological parameters, timely initiation is crucial for reversing severe metabolic derangements and facilitating a more rapid patient recovery [14,22].

In the univariate model, we demonstrated clinical symptoms, particularly altered consciousness, oliguria, and respiratory failure associated with increased mortality in patients with MALA–AKI. The present of alteration of consciousness is increase mortality risk which consistent with the study by Siangtrong et al. [20] Several factors significantly associated with higher mortality included low serum bicarbonate, lower blood pH, high serum lactate, elevated SOFA score, prolonged PT, lower hemoglobin and use of vasoactive medications [14], however, HD associated with lower mortality when compared to other KRT modalities. This study found that blood pH levels were significantly lower in non-surviving patients, particularly those with a pH < 7.0. This finding is consistent with previous studies by Moioli et al. which reported significantly lower pH in non-survivors than survivors [23]. However, some studies have found no significant association between blood pH and mortality [11,12,19]. In the study by Kajbaf and Lalau [12], which focused specifically on patients with severe acidosis (pH < 7.0), the pH levels between the survivor and non-survivor groups did not differ significantly, potentially explaining the lack of a significant association. Additionally, our study found that lower blood bicarbonate levels were associated with higher mortality rates, particularly in patients with bicarbonate levels <5 mmol/L in univariate analysis, though this association was not statistically significant in the multivariate analysis. Some other studies have likewise not identified a significant relationship between blood bicarbonate levels and mortality [18,19]. Our study found that higher lactate levels were associated with higher mortality in univariate analysis. This finding is consistent with previous studies [19,22]. However, some studies found no association between lactate levels and mortality [11,24]. This may be due to the small sample sizes in these studies.

In the multivariate analysis, oliguria, prolonged PT, and a higher SOFA score remained independently associated with higher in-hospital mortality, and HD was independently lower in-hospital mortality in MALA–AKI patients. The association between prolonged prothrombin time and higher mortality may indicate worsening liver function or may be a consequence of shock [11]. Our study underlined oliguria independently associated with higher mortality in MALA–AKI patients, which may be explained by oliguria reflecting the severity of renal impairment leading to higher mortality. The SOFA score is a validated system for classifying disease severity linked to adverse clinical outcomes in critically ill patients [25,26], especially higher SOFA scores were significantly associated with the severity of lactic acidosis in MALA patients [23]. Our findings align with previous studies, which have shown that a higher SOFA score is independently associated with an increased risk of in-hospital mortality [25,26].

Our study revealed that KRT via HD had the lowest mortality rate, followed by continuous kidney replacement therapy (CKRT) compared with APD. Previous studies have shown that MALA patients who received HD had lower mortality rates than those who received APD; however, this difference did not reach statistical significance in a systematic review of case reports and case series [13,17,19,27]. Additionally, the number of patients who received APD treatment was small [19]. The previous studies indicate that CKRT can be used in the treatment of MALA–AKI to reduce mortality [15,16,28]. However, recent studies have found that MALA patients who underwent HD had a significantly lower mortality rate than those treated with other dialysis modalities [13,29], which is consistent with our findings. Our result supports the Extracorporeal Treatment for the Metformin Poisoning guidelines (EXTRIP) guideline, which recommends intermittent HD as the first-line treatment for metformin poisoning [14], as well as with the KDIGO clinical practice guideline for AKI, which recommends KRT in cases of life-threatening acid-base or electrolyte disturbances that are refractory to medical management [21]. Furthermore, the EXTRIP recommendations suggest using CKRT in cases where HD is not feasible or in patients with hemodynamic instability, similar to the KDIGO guideline [14,21]. In line with these recommendations, HD appears to be the most efficient therapy in MALA–AKI due to its superior clearance of metformin and lactate, whereas CKRT is preferred when hemodynamic instability precludes intermittent therapy. Theoretically, metformin, with a molecular weight of 129 Daltons, is widely recognized as dialyzable due to its high water solubility and low protein binding. These properties allows for elimination through KRT, particularly diffusive treatment, which is more effective than peritoneal dialysis in removing metformin and lactic acid and reducing metabolic acidosis [14,23,30,31].

To our knowledge, this study represents one of the largest cohorts of MALA–AKI (*n* = 143) in Southeast Asia to date. A unique aspect of our work is the direct, head-to-head comparison of outcomes for patients treated with HD, CKRT, and APD, providing insights not available in smaller case series. The primary results indicated that the mortality rate among MALA–AKI patients was high, and KRT was associated with reduced in-hospital mortality. Among the KRT modalities, HD treatment was associated with the lowest mortality in this setting.

Our study has some limitations. Firstly, due to its retrospective design, certain confounding factors and selection bias may have influenced the study outcomes. Thus, multivariable analysis was performed to minimize the impact of potential confounding factors and baseline differences. Secondly, this study did not consider plasma metformin concentration as part of the diagnostic criteria for MALA due to the lack of available testing and its impracticality in routine clinical settings. However, we ensured the exclusion of sepsis and other potential causes of lactic acidosis. Thirdly, since most patients in this study were classified as AKI stage 3, thus initiating dialysis earlier before reaching this stage should be carefully considered.

In conclusion, MALA–AKI was associated with poor outcomes and a high mortality rate. KRT led to a lower mortality rate than supportive care, with HD emerging as the most effective treatment modality. Early KRT initiation (<6 h) showed a trend toward lower mortality while oliguria, prolonged prothrombin time, and higher SOFA scores were identified as independent prognostic markers. Comprehensive, well-designed studies on a larger scale are required to validate these promising findings.

## Data Availability

The datasets used and/or analyzed during the current study are included within the article.
